# Scoping review of non-pharmacological or self-management interventions for perinatal mood and anxiety disorders tested with Hispanic and Latina women

**DOI:** 10.3389/fpsyt.2025.1644798

**Published:** 2025-12-05

**Authors:** Leslie B. Lantz, Allison A. Gregory, Roy Brown, Susan M. Bodnar-Deren, Kim A. Case, Patricia A. Kinser

**Affiliations:** 1 School of Nursing, Virginia Commonwealth University, Richmond, VA, United States; 2 Libraries, Virginia Commonwealth University, Richmond, VA, United States; 3 Department of Sociology, Virginia Commonwealth University, Richmond, VA, United States; 4 Department of Psychology, Virginia Commonwealth University, Richmond, VA, United States

**Keywords:** cognitive behavioral therapy, cognitive behavioral stress management, mindfulness, relaxation, depression, anxiety, non-pharmacological, perinatal mood and anxiety disorders

## Abstract

**Introduction:**

Research on ethnic disparities in perinatal mental health disorders among Latina and Hispanic women has yielded mixed findings. This scoping review aims to identify and describe non-pharmacological or self-management interventions for preventing or reducing symptoms of depression, anxiety, and/or stress associated with perinatal mood and anxiety disorders (PMADs) in Hispanic and Latina women.

**Materials and methods:**

We used the Joanna Briggs Institute (JBI) scoping review methodology and conducted literature searches in MEDLINE (OVID), Embase (OVID), Web of Science, CINAHL, and PsycINFO (EBSCO). Terms such as “self-management interventions,” “mindfulness,” “relaxation,” “exercise,” and “mind-body therapies” were tailored to each database. Additionally, we included the titles of specific interventions that arose in the original searches. We included globally inclusive full-text primary studies published in English or Spanish in peer-reviewed journals, without restrictions on publication date. Studies met the inclusion criteria if they evaluated non-pharmacological or self-management interventions, including mind-body complementary therapies for preventing or reducing symptoms such as depression, anxiety, and/or stress associated with PMADs in predominantly Hispanic or Latina pregnant or postpartum samples.

**Results:**

Twelve studies met inclusion criteria, evaluating a non-pharmacological or self-management intervention in predominantly Hispanic or Latina pregnant or postpartum study samples. The twelve studies evaluated four interventions that used principles of cognitive behavioral therapy (CBT), cognitive behavioral stress management (CBSM), mindfulness, or relaxation. In general, most studies found that the intervention was either equally as effective or more effective at reducing depression, anxiety, or stress symptoms or major depressive episodes as the control group. However, due to the use of varied outcome measures and follow-up timelines, additional research employing consistent RCT methodology is recommended. This would allow for a comprehensive systematic review and meta-analysis to be conducted to determine the effectiveness for these populations.

**Discussion:**

We identified studies in which CBT, CBSM, mindfulness, and relaxation interventions were tested with a study population of predominantly Hispanic and Latina perinatal women. Our findings suggest that these non-pharmacological or self-management interventions should receive more research attention given their potential effect on the prevention or reduction of symptoms such as depression, anxiety, and/or stress in Hispanic and Latina perinatal women.

## Introduction

Throughout pregnancy and up to the first year postpartum (i.e., the perinatal period), mental health symptoms and disorders, including depression, anxiety, and/or stress, are often referred to as perinatal mood and anxiety disorders [PMADs; (1)]. They are common morbidities and collectively pose a major threat to public health (2, 3). These include symptoms of depression, anxiety, and/or stress associated with PMADs (1). Each disorder within the PMAD umbrella is diagnosed based on the DSM-5-TR criteria (4). For example, perinatal depression, one of the most significant perinatal complications, affects between 10-20% of individuals in the United States (5, 6). Perinatal depression symptoms can range from mild to severe, with well-documented adverse outcomes for women and their infants including reduced maternal self-efficacy (7), preterm birth (8), and increased suicidal ideations (9). The prevalence of women who experience perinatal anxiety is between 13% and 21% (10), and perinatal stress is between 6% and 78% (11). Despite the prevalence of perinatal mental health symptoms and disorders, they are often unrecognized, untreated, or undertreated (12).

Particular concerns include disparities in the prevalence rates of perinatal mental health disorders in the U.S., based on racial and ethnic differences, with minoritized women experiencing higher rates of PMADs compared to White women (13). The Hispanic and Latina populations represent 20% of the U.S. population, second to non-Hispanic White people. Between 2022 and 2023, the Hispanic and Latina population grew faster than any other group, primarily due to Hispanic and Latina births (14). Among Hispanic and Latina women, being of low socio-economic status, having elevated stress, and poor social support increases the risk of perinatal depression and anxiety (15, 16). For example, chronically stressed Hispanic and Latina women were less likely to be healthy during pregnancy compared to their non-Hispanic White counterparts (17). A recent systematic review found higher rates of postpartum depression (24%) compared to the general population (12-17%), and rates of anxiety were 19% for Hispanic and Latina women, compared to 15% in the general population. Other perinatal mental health problems are more prevalent in migrants compared with non-migrants (18).

Research on ethnic disparities in perinatal mental health disorders among Hispanic and Latina women has yielded mixed findings. Some studies reported a higher prevalence of PMADS among Hispanic and Latina women compared to non-Hispanic White women (13), while others found no significant differences or even lowered risk, depending on factors such as nativity, acculturation, and socioeconomic context (16, 19). These discrepancies may reflect important heterogeneity within Hispanic and Latina populations, including differences by immigration status, country of origin, and cultural or linguistic barriers (20, 21). However, a body of research found that Hispanic and Latina women are at higher risk for PMADs compared to their non-Hispanic or Latina counterparts (13). This is salient amongst individuals not born in the U.S (20). due to unique psychosocial experiences such as increased acculturation (22). Among individuals not born in the U.S., rates of experiencing perinatal mental health disorders are not equally distributed across migrant population subgroups. In a recent meta-analysis of migration and perinatal mental health, Fellmeth and colleagues (20) found that PMADs affect approximately one-third of migrant women; however, rates differ by country of origin and destination, language and cultural barriers, social isolation, and support. In addition to biological changes experienced by women during pregnancy (23), research suggested that the social determinants of health (SDoH) played a major role in perinatal mental health outcomes (24). Addressing the SDoH is a public health priority and the foundation of Healthy People 2030 (25). The SDoH are non-medical factors influencing health outcomes such as the conditions in which people are born and live, socioeconomic status, immigration status, and racism or discrimination, and play a major role in individuals’ health outcomes (26). Given the high risk of perinatal mental health disorders among Hispanic and Latina populations, evidence for low-cost interventions is urgently needed, particularly those which are non-pharmacological [i.e., non-drug therapies which could include psychological interventions; (27)] or involve self-management strategies [i.e., interventions encouraging active engagement in behaviors to improve one’s health; (28, 29)].

Since perinatal depression and anxiety are among the most common complications of pregnancy and postpartum and an important contributor to pregnancy-related deaths, there is a public health need for pragmatic, low-cost, non-pharmacological interventions to prevent or reduce perinatal mental health disorders and symptoms and promote self-management strategies (30, 31), particularly those deemed to be culturally and linguistically appropriate by Hispanic and Latina populations. Treatments for PMADs are available (e.g., therapy, support groups, online courses), yet underutilized, especially among racialized and ethnic populations (7, 32). Preferences about various types of treatments differ amongst ethnic subgroups, with minoritized women more likely to opt for non-pharmacological interventions. For example, while Hispanic and Latina women are less likely than other ethnic subgroups (e.g., Black/African American, Asian or Pacific Islander, Indigenous, or Arab/Middle-Eastern/North African) to seek treatment for PMADs, when they do seek care, they are more likely than non-Hispanic White women to seek non-pharmacological approaches (33). These preferences may reflect concerns about potential side effects of medication during the perinatal period (34), cultural stigma surrounding mental health diagnoses (35), and limited access to linguistically and culturally concordant providers (21). In addition, Rokicki and colleagues (34) emphasized that perceived and experienced discrimination, low trust in providers, and lack of culturally responsive care were among the key barriers to accessing mental health support, particularly for immigrant women.

A small body of evidence supports non-pharmacological (e.g., cognitive behavioral therapy) or self-management interventions (e.g., mindfulness) for prevention or management of perinatal mental health disorders (36, 37); however, few studies focused on minoritized women (30). When considering extant literature exploring these interventions and whether they may reduce perinatal depression, anxiety, and/or stress symptoms, a number of systematic reviews have been conducted. For example, Li and colleagues (38) analyzed 79 randomized and quasi-randomized controlled trials of cognitive behavioral therapy (CBT) in perinatal populations, finding that CBT was effective in reducing depression, anxiety, and stress in both the short and long term. Lever Taylor and colleagues (39) conducted a meta-analysis of 17 studies on mindfulness-based interventions in perinatal populations and found small to moderate improvements in depression, anxiety, and stress, but no significant benefits over control groups, likely due to methodological limitations and a focus on non-clinical, primarily antenatal samples. Urech and colleagues (40) conducted a systematic review and meta-analysis of 32 studies involving 3,979 pregnant women and found that relaxation interventions (e.g., yoga, music, breathing, mindfulness) significantly reduced maternal stress, anxiety, and depression.

However, in each of the systematic reviews with meta-analyses, the literature does not discuss their findings in the context of predominantly Hispanic or Latina perinatal populations. Therefore, we have conducted a scoping review to identify and describe non-pharmacological or self-management interventions for preventing or reducing symptoms such as depression, anxiety, and/or stress associated with PMADs in Hispanic and Latina women populations.

## Materials and methods

We utilized the Joanna Briggs Institute (JBI) scoping review methodology to conduct our review (41). We adhered to the Preferred Reporting Items for Systematic Reviews and Meta-Analyses extension for Scoping Reviews (PRISMA-ScR) guidelines to write the report (42). We used the population, concept, and context framework (41) to craft the review question: What non-pharmacological or self-management interventions for preventing or reducing symptoms of depression, anxiety, and/or stress associated with perinatal mood and anxiety disorders PMADs have been tested and published in Hispanic and Latina women?

### Search strategy

We systematically searched globally inclusive published, peer-reviewed literature for primary studies evaluating non-pharmacological or self-management interventions for Hispanic or Latina women experiencing symptoms such as depression, anxiety, and/or stress associated with PMADs. We searched the following databases: MEDLINE (OVID), Embase (OVID), Web of Science, Cumulated Index to Nursing and Allied Health Literature (CINAHL), and PsycINFO (EBSCO), from inception through May 21, 2024. We did not restrict publication dates to retrieve as many reports as possible. Keywords and controlled vocabulary terms related to “non-pharmacological interventions,” “self-management,” “mindfulness,” “relaxation,” “exercise,” and “mind-body therapies,” were tailored to each database as well as incorporating the titles of specific interventions that arose in the original searches. The search strategy is provided (see **Supplementary Table S1**). References of relevant publications were hand-searched to identify additional publications.

### Inclusion criteria

The eligibility criteria were based on JBI’s population, concept, and context framework (41). We included full-text reports of qualitative and quantitative primary studies published in English or Spanish, in peer-reviewed journals, where the study samples included 51% or greater Hispanic or Latina participants in the perinatal period, who were 18 years or older. To be included, the studies had to evaluate non-pharmacological and/or self-management interventions for preventing or reducing symptoms such as depression, anxiety, and/or stress associated with PMADs. We excluded reports if there were fewer than 51% Hispanic or Latina perinatal participants or if the full study population was less than 18 years of age.

### Study selection

**Figure 1:**
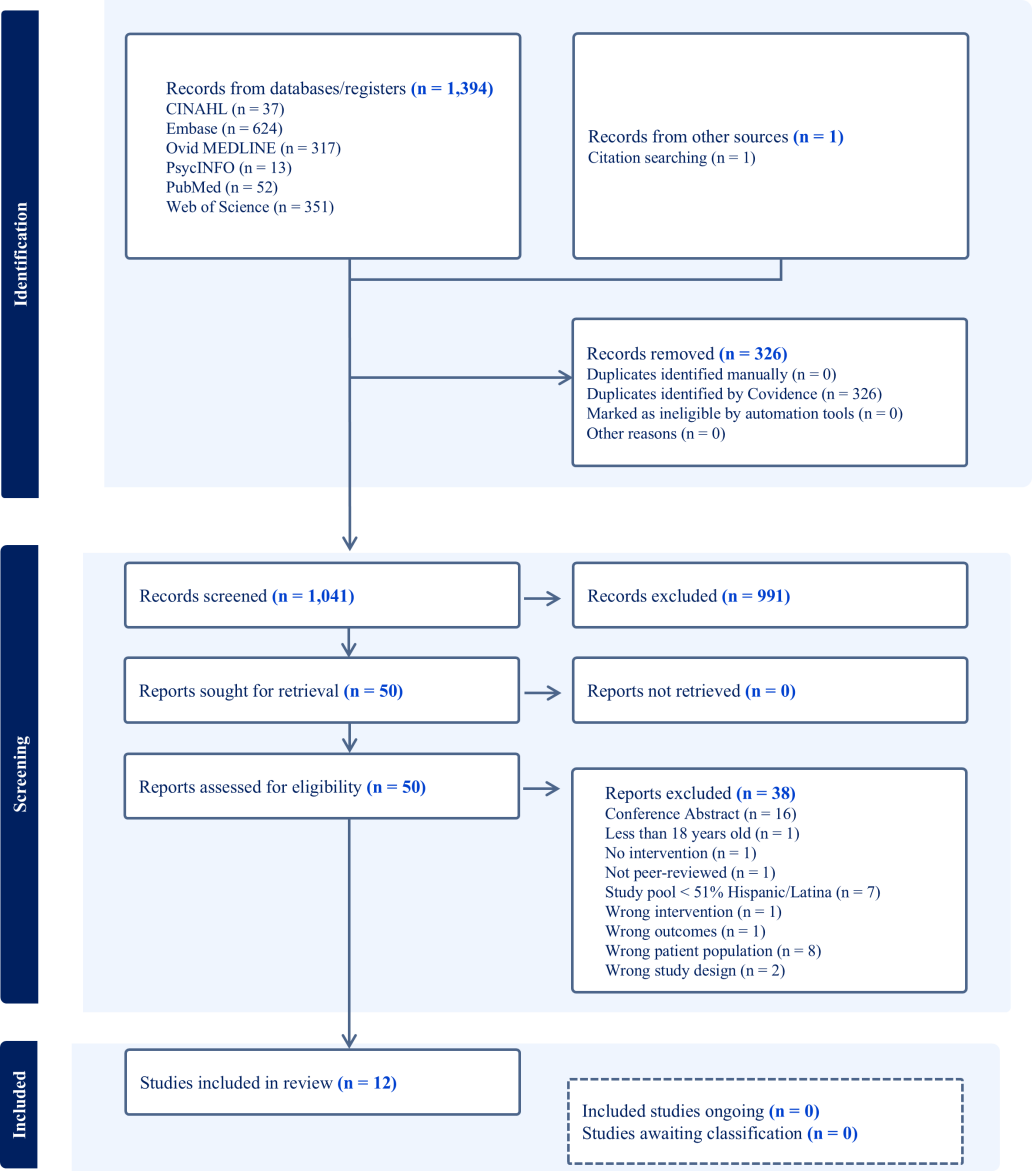
Flowchart depicting the literature search and selection process.

We used the Covidence systematic review management software (43), which permits independent screening and blinding of decisions by team members and adheres to a systematic, multiphase selection review process (44). We included the results of our screening and selection review processes in a Preferred Reporting Items for Systematic Reviews and Meta-analyses (PRISMA) flow diagram (45) in **Figure 1**. After removing 326 duplicates, we screened the titles and abstracts of 1,041 records. Two reviewers (LL and AG) independently screened all the titles and abstracts, and one reviewer (PK) resolved any existing conflicts. After screening all titles and abstracts, 50 reports remained for full-text review. During this process, two reviewers (LL and AG) independently screened all full-text reports, and one reviewer (PK) resolved any existing conflicts. We excluded 38 full-text reports and recorded the exclusion reasons in the PRISMA flow diagram (see **Figure 1**). After we eliminated these reports, 12 studies met the full eligibility criteria for further evaluation.

### Data extraction and synthesis

We extracted the following data from the included reports: author(s), year of publication, study design and purpose, country of origin, intervention, intervention and control groups, cultural adaptation, population and follow-up, ethnicity, demographics, outcomes, and measures. We organized the data extracted into a table (see **Supplementary Table S2**) and narratively summarized the reports according to the identified interventions. The data were organized into a table (see **Supplementary Table S2**) to identify and describe types of non-pharmacological or self-management interventions, including CBT, CBSM, mindfulness, and relaxation. In the outcomes and measures column, the degree of intervention effectiveness is indicated with codes (e.g., intervention effective or ineffective) along with specific measures used in the study (see **Supplementary Table S2**). We acknowledge that particular groups may prefer “Hispanic,” Latina,” or a more specific ethnicity. When reporting outcomes for specific studies, we default to the term(s) that the original authors used to describe the participants in their studies.

## Results

The 12 studies were published from 2007 to 2024. Nine studies were conducted in the U.S (46–54)., one in Brazil (55), one in Chile (56), and one spanned 23 countries worldwide with most participants in Chile (18.9%), Spain (16.2%), Argentina (11.7%), and Mexico (10.8%) (57). Of the studies in the U.S., four studies focused on specifically recruiting Hispanic or Latina women (46–49) while five studies focused on low-income women and ended up recruiting a majority of Latina women in their samples (50–54).

The most common study design was a randomized controlled trial (RCT) (47, 50–54, 56, 57), followed by three quasi-experimental studies (46, 49, 55) and one secondary qualitative analysis from Le and colleagues’ 2011 RCT (48). The studies included 1,054 total participants with sample sizes ranging from 39 to 217. For 9 of the 12 studies, between 36% and 100% of participants completed less than a high school education. For 8 of the 12 studies, participants reported annual household incomes less than $30,000. Nine studies focused on pregnant women mostly in prenatal care settings. Seven studies included CBT-based interventions: one study used the Mamá, te entiendo program, and six studies used the Mothers and Babies Course. Three studies included the same cognitive behavioral stress management intervention— the Stress Management and Relaxation Training for Moms program. One study included a mindfulness intervention— the Centering Pregnancy with Mindfulness (CP+) program. One study used a non-manualized relaxation technique program. Preliminary evidence regarding non-pharmacological or self-management interventions, including CBT, CBSM, mindfulness, and relaxation, may be associated with preventing or reducing symptoms such as depression, anxiety, and/or stress associated with PMADs.

Five studies focused on reducing symptoms: one for depression, anxiety, and stress (46), one for stress (52), one for anxiety (54), and two for depression (56, 57). Five studies focused on preventing symptoms of depression (47, 49) including symptoms that reached the threshold of the definition of major depressive episodes (MDEs) (50), depression and stress (53), and anxiety (55). One study explored the effect of intervention fidelity on outcomes (51), and another elucidated the lack of difference between RCT intervention and control groups (48).

Measures varied across the included studies (see **Supplementary Table S2**). Identified in seven studies, perinatal depression was the most common measure. The most common measures were the Center for Epidemiologic Studies Depression Scale [CES-D; (58)] (46, 47, 50, 53) and the Edinburgh Postnatal Depression Scale [EPDS; (59)] (46, 50, 56, 57). The Beck Depression Inventory- Second Edition [BDI-II; (60)] (47), Postpartum Depression Screening Scale- Short Form [PDSS-SF; (61)] (49), and the 9-item Patient Health Questionnaire [PHQ-9; (62)] (56) were each used once. Three studies measured anxiety; the State-Trait Anxiety Index [STAI; (63)] was used in two studies (46, 55), and the State-Trait Personality Inventory- Short Form [STPI-SF; (64)] and Pregnancy Related Anxiety Scale [PrAS; (65)] were used in the remaining study (54). Three studies assessed stress using various measures such as a visual analog scale (53), the 14-item Perceived Stress Scale [PSS-14; (66)] (52), and the 10-item Perceived Stress Scale [PSS-10; (66)] (46). Urizar, Caliboso, and colleagues (51) assessed positive and negative mood states using the Positive and Negative Affect Schedule [PANAS; (67)] (53) and the Positive and Negative Affect Schedule- Short Form [PANAS-SF; (68)] (51). Major depressive episodes were measured using a Mood Screener [Mood Screener; (69)] (47, 50, 57).

### Cognitive behavioral therapy interventions

Seven studies included interventions based on principles of CBT used during the perinatal period. Six of these studies exclusively used the Mothers and Babies Course (MBC), a program developed by Dr. Muñoz and colleagues (50), focused on preventing depression symptoms, including those meeting the threshold for MDEs. While each of these studies evaluated the same intervention program (MBC), they were conducted with different participant samples (e.g., in separate geographic locations or populations) and are therefore treated as distinct studies in the synthesis. Barrera and colleagues’ (57) study transitioned the MBC program from an in-person to an Internet-delivered format. Three of the seven MBC studies included Le as the primary author. The Le, Perry, and Stuart (47) parent study was followed by a qualitative exploratory study with a subset of participants (48) to understand the lower than anticipated depression rates in the control and intervention groups, whereas Le and colleagues (49) used a distinct sample. A subset of participants from the Muñoz and colleagues’ (50) study were also included in the expanded study by Urizar and Muñoz (53). The seventh CBT-based study was conducted by Franco and colleagues (56) using the Mamá, te entiendo web app, a program developed by Franco and colleagues (70). Four of the CBT-based RCTs detected trends in decreased depression over time in both groups as well as minimal MDE occurrences (see **Supplementary Table S2**).

Barrera and colleagues (57) conducted a pilot RCT comparing an internet-based MBC program with eight sequential, independently completed lessons with an active control (access to an online postpartum depression brochure). Their completely remote study included pregnant women (*n* = 92; 82.9% Spanish-speaking) residing in 23 countries worldwide. Preliminary analysis revealed that both groups experienced a decrease in depression symptoms over time, with no significant difference by group in depression symptoms over time.

In the Le and colleagues (47) RCT, MBC was offered as 2-hour, weekly sessions over eight weeks to healthy pregnant women at risk of depression; the majority of the study population were U.S. immigrants born in Central and South America or Mexico. Those randomized to the MBC (*n* = 112) had a short-term decrease in depression symptoms (from baseline to post-intervention) as compared to usual care (*n* = 105), yet the group differences did not persist into the postpartum period. The authors evaluated rates of symptoms meeting the criteria for MDEs in both groups and found there to be no difference in cumulative incidence during the study period (up to 12 months postpartum). Subsequently, to understand the lack of treatment-effect findings, Le and colleagues (48) conducted a qualitative exploration (*n* = 39) into the experiences of a subset of the RCT participants. Participants in the prior intervention group reported that the course reduced the effect of stress on their moods and thoughts. Participants in the prior usual care control group identified their relationships with the research team as a factor in decreasing their sense of isolation and improved maternal self-efficacy. The usual care control group participants had increased awareness about their moods, mood-regulation skills, and sources of social support. Participants in both groups reported that engaging in the study was beneficial in various ways.

Le and colleagues (49) evaluated an abbreviated version of the MBC (six weeks) using a non-randomized, uncontrolled, mixed methods design (*n* = 86; 76.7% Central American immigrants) with pregnant or postpartum women at high risk for depression. A comparison between completers, non-completers (partial participation), and those who completed no classes demonstrated that all participants experienced a decrease in depression symptoms, with no difference between the groups over time. Of note, no participants had elevated symptoms meeting the diagnostic criteria for MDEs at three months post-intervention.

Muñoz and colleagues (50) conducted an RCT comparing a 12-week MBC with usual care in *n* = 41 healthy low-risk pregnant women at high risk for developing elevated symptoms of depression meeting the threshold for MDEs. Seventy percent were Spanish-speaking Latina women born in Mexico or Central America. The findings from this study and a subsequent analysis of a subset of participants (*n* = 24; 80% Spanish-speaking) by Urizar and Muñoz (53) suggest that there were no significant group differences over time in depression or stress symptoms; however, participants in the MBC experienced fewer symptoms of MDEs than those in the control group, albeit with a small effect size.

Franco and colleagues (56) conducted an RCT using an eight-week self-paced internet-based program with guidance from a clinical psychologist. Their study occurred in Chile and included postpartum women with minor or major depression symptoms (*n* = 55; 93.8% Chilean), comparing the Mamá, te entiendo app-based program (*n* = 33) versus a usual care wait-list control (*n* = 32). Although the main focus of findings centered on feasibility and acceptability, preliminary analysis revealed that both groups experienced a decrease in depression symptoms over time, with no significant difference by group in depression symptoms over time.

### Cognitive behavioral stress management intervention

Three studies focused on an intervention called SMART Moms, developed by Urizar and colleagues (71), based on principles of CBSM (51, 52, 54). This intervention involves a group-based facilitator-led session with two-hour sessions over eight weeks. Two of the studies were from a single RCT (51, 52) evaluating the effect on reducing perceived stress and a secondary study evaluating the effects of treatment fidelity on outcomes. One study (54) focused on reducing anxiety symptoms. None of the CBSM studies focused on the prevention of symptoms.

Ponting and colleagues (54) conducted an RCT comparing the SMART Moms intervention with an active-control education group among healthy, low-risk pregnant women (*n* = 100) who self-identified as either Latina (74%) or Black (18%). Ponting and colleagues’ findings suggest no main effect of intervention or time in state- or pregnancy-specific anxiety between groups, but may have been confounded by potential effects of the education provided to the control group.

Two publications reported on various stress findings from a single RCT comparing the SMART Moms intervention (*n* = 55) with an active-control education group (*n* = 45), in a healthy low-income pregnant population (71% Latina) (51, 52). Urizar and colleagues report significant decreases in a study-specific stress measure and Perceived Stress Scale scores over time compared to the active control group.

### Mindfulness and relaxation interventions

Two studies focused on interventions based on other principles. One included a mindfulness-based intervention during the perinatal period from the Mindfulness-Based Childbirth and Parenting (MBCP) program (46), developed by Bardacke (72). Another included a relaxation intervention, developed by Benson (73), used during the postpartum period (55).

Duncan and colleagues (46) conducted a non-RCT longitudinal study with healthy low-risk pregnant women (*n* = 49), 65% of whom were Latina; 63% of the Latina women were Spanish-speaking. They participated in an active control group-based Centering Pregnancy (CP) program or Centering Pregnancy enhanced with mindfulness skills (CP+) from the MBCP program. The CP+ group had lower rates of postpartum depression over time than did the CP group. There were trends toward greater decreases in perinatal anxiety in the CP+ group than in CP alone. There were no significant group differences, as both groups had slightly decreased symptoms over time. Greater improvements in these scores were detected among certain participants in the intervention group with lower baseline stress and depression scores.

Primo and Amorim (55) conducted a non-RCT in Brazil comparing a relaxation-based intervention (non-manualized relaxation techniques twice daily for two consecutive days) to usual care for healthy patients at most two days postpartum with *n* = 60 in the hospital setting. They found that from baseline to one week postpartum, the intervention group experienced a decrease in anxiety symptoms compared to the control group. The authors did not provide details about the participants’ ethnicities.

## Discussion

This scoping review suggests that, based on the findings in the 12 studies, interventions based on principles of CBT may help prevent or reduce depression symptoms; however, they may not help prevent stress symptoms. Interventions based on the principles of CBSM may help reduce stress symptoms in the short term; however, they may not help reduce anxiety symptoms. Mindfulness-based interventions may help reduce depression or anxiety symptoms; however, interventions may not be helpful in persistently reducing stress symptoms. Relaxation may help prevent anxiety symptoms (see **Supplementary Table S3**). However, it is difficult to generalize findings about the efficacy of mindfulness-based or relaxation interventions, as each was only explored in one study with the target population (predominantly Hispanic or Latina perinatal individuals). Based on the heterogeneous methodological approaches, various interventions used in these studies, and mixed results, determining the precise mechanisms responsible for effectiveness or lack thereof for various outcomes remains difficult. **Supplementary Table S3** provides an overarching summary of the scoping review findings across the studies that were included. The current state of the science is that a small group of researchers intentionally focus on these particular topics within Hispanic and Latina populations. Future research and systematic reviews with meta-analyses are likely warranted to determine the effectiveness of these interventions in this population.

The mechanisms by which these interventions may have had impact on mental health symptoms in the study populations cannot be fully hypothesized and tested within this scoping review. However, given the state of the science regarding these interventions, it may be reasonable to suggest that two elements warrant further exploration: First, the degree to which an intervention was adapted for cultural congruence and/or linguistic adaptability may be important yet it is unclear whether/how this can differ between an intervention and control group. As seen in **Supplementary Table S2**, most studies did not discuss the degree to which cultural congruence or linguistic (e.g., terminology) adaptability was entailed in their interventions. Of the five CBT-based MBC intervention studies (47–50, 53) and the one mindfulness-based MBCP study (46) that included these details, no significant between-group differences were detected in preventing or reducing symptoms. Second, the extent to which participants may have experienced social support through the intervention and control groups may be important. The majority of the studies evaluated interventions based on cognitive behavioral techniques, either CBT or CBSM, in group-based formats. This may reflect the importance of social support in this population, as noted in a qualitative study of treatment preferences in a study that included Latina mothers (21). Of the five MBC intervention studies (47–50, 53), three SMART Moms intervention studies (51, 52, 54), and MBCP program study (46), the only studies in which there were statistically significant between-group differences were the two Urizar and colleagues’ studies (51, 52) in which the control group only received printed educational materials (i.e., there were no social interactions between participants). Le and colleagues (48) posit that the benefits of perceived social support in their control group participants may have been responsible for the lack of between-group differences.

Overall, the non-pharmacological and self-management interventions evaluated in this scoping review are in alignment with those that have been evaluated in larger populations without respect to Hispanic or Latino culture or ethnicity (86); notably, CBT is a very commonly-studied non-pharmacological intervention for many conditions and populations and was highly represented in this review. The majority of the CBT-based RCTs found trends in decreased depression symptoms in both the intervention and active control groups, and these findings are consistent with longstanding broader research evidence that has established the efficacy of CBT in treating depression (74). Despite the lack of statistical significance between groups, Le and colleagues’ (48) qualitative study revealed that interviewed participants from the previous study’s control group experienced an increased awareness of social support, improved maternal self-efficacy, and decreased sense of isolation. This is consistent with other intervention-based literature in which some control groups experienced similar benefits to the intervention group, through social interactions and experiences facilitated by the study (75–77). In addition, Li and colleagues’ (38) systematic review and meta-analysis found that CBT-only and CBT in conjunction with other interventions yield short- and long-term decreases in depression symptoms and short-term efficacy in reducing stress symptoms. These findings differ from those in a RCT with perinatal women in which the intervention group experienced significantly lower depression and stress symptoms compared to the control group (78).

Of the three studies evaluating interventions based on principles of CBSM, two studies (reporting on a single RCT) detected significant, persistent decreased stress symptoms over time in the intervention group compared to the active control group. However, one study found the intervention did not result in significant persistent differences in anxiety symptoms over time as compared to an active control group. The state of the science about CBSM is less evolved than that of CBT; the three recently-published systematic reviews that include CBSM have focused on populations with HIV, chronic fatigue syndrome, and breast cancer, all of which suggest that perceived stress can be decreased after a CBSM intervention but that methodological rigor must be addressed (79–81). A lack of persistent differences in anxiety and stress symptoms over time mirrors findings in an RCT in which an internet-based CBSM intervention was utilized (40). These findings differ in an RCT with prenatal women in which both groups experienced significant decreases in anxiety and stress symptoms over time (82). A significant persistent decrease in stress symptoms between groups is consistent with findings in an RCT which found stress significantly decreased post-intervention in the intervention group while stress increased in the control group. Two weeks post-intervention, a significant difference in stress symptoms between groups remained (83).

Despite that there is a significant body of literature around mindfulness-based interventions for a variety of populations including pregnant individuals (84), very few studies have focused on the intervention for this target population. Included in this review was a study of a mindfulness-based intervention in a Hispanic and Latina perinatal sample by Duncan and colleagues (46); findings of the Duncan study were consistent with those reported in Lever Taylor and colleagues’ (39) systematic review and meta-analysis in which mindfulness-based interventions in the perinatal period had moderate pre-post effects in depression symptoms and smaller effects related to anxiety symptoms. Additionally, in a non-RCT with a low-income racially heterogeneous U.S. sample and inclusive of an active control group and three mindfulness-based intervention groups (one prenatal, one postpartum, and one postpartum parenting group), researchers found a decrease in depression symptoms with a trend in lower anxiety symptoms for those in the prenatal intervention group, albeit the decrease in depression symptoms did not persist through the postpartum period; the postpartum parenting group experienced decreases in anxiety symptoms compared to all other groups (85). These findings differ from those in an RCT pilot study of mothers with at least one marginalized identity that used mindful prenatal yoga with an active control group and found statistically significant decreases in depression, anxiety, and stress symptoms between groups over time (86).

In this scoping review, only one study was found that tested a relaxation intervention with the population of interest: Primo and Amorim’s (55) study evaluating a relaxation intervention found a decrease in anxiety symptoms in the intervention group compared to the usual care group. Of note, the intervention was delivered in the hospital setting in Brazil (during childbirth and the early postpartum period). These findings are consistent in an RCT using relaxation training in which significant decreases in anxiety and stress symptoms were found in the intervention group compared to the control group (usual prenatal care) (87). Abera and colleagues (88) found in their systematic review and meta-analysis that prenatal relaxation interventions (e.g., yoga, music, Benson relaxation, progressive muscle relaxation, deep breathing relaxation, guided imagery, mindfulness and hypnosis) resulted in overall decreased depression, anxiety, and stress symptoms. These findings, however, differ from Yu and colleagues’ (89) systematic review which focused on non-pharmacological interventions in women with high-risk pregnancies, in which relaxation interventions did not improve anxiety symptoms. In that review, two studies found a significant reduction in anxiety symptoms; however, three studies found no significant difference in anxiety symptoms.

## Limitations

We acknowledge four limitations to our scoping review. First, we included research from various countries, yet findings should not be considered generalizable to all Hispanic and Latina women. Second, we included globally inclusive studies published in both English and Spanish; however, relevant studies may have been published in other languages. Third, interventions without full manualization may be difficult to compare. Fourth, we did not include grey literature (e.g., conference abstracts & papers, white papers, dissertations, etc.) as we limited our search to primary studies published in peer-reviewed journals.

## Future research directions

There are several key takeaways unique to the perinatal mental health experiences of Hispanic and Latina women regarding the use of non-pharmacological or self-management interventions. The variability in duration of studies with subsequent baseline assessments may be important, considering the perinatal period extends to twelve months postpartum. Considering 54.5% of the studies had follow-up assessments at twelve months or greater during the postpartum period, there is an opportunity to conduct more longitudinal RCTs, inclusive of a minimum of twelve months postpartum. Howard and Khalifeh (90) discussed how the evidence supports the importance of perinatal mental health interventions longitudinally, such as during the first 1,001 days of the infant’s life (from conception to two years of age). There is a need for more robust RCTs, inclusive of non-pharmacological or self-management interventions centered around perinatal mental health for predominantly Hispanic and Latina women. Rokicki and colleagues (34) discussed the critical need for improving access to mental health interventions and programs for racial and ethnic minorities to increase perinatal mental health equity. Future studies could explore which specific components of the interventions may be particularly beneficial for Hispanic and Latina women, such as through mechanistic clinical trials as well as through qualitative inquiry, as well as attend to underlying cultural, linguistic, or socioeconomic factors. Improved treatment effects may occur in control groups similar to benefits experienced in the intervention group (77). Le and colleagues (48) explored this phenomenon through a secondary study from a primary RCT study, and consideration should be given to conducting mixed methods or secondary studies of a similar nature to explore this phenomenon.

### Implications for healthcare professionals

The findings of this scoping review may increase awareness among healthcare professionals of existing interventions tested among Hispanic and Latina women experiencing symptoms of depression, anxiety, and/or stress associated with PMADs. Being aware of these non-pharmacological or self-management interventions may be imperative to supporting or delivering patient care. Additionally, these interventions are supported by groups such as the American College of Obstetricians and Gynecologists (ACOG), as they suggest psychoeducation, psychotherapy including CBT and CBSM, mindfulness, and relaxation for the management of perinatal mental health conditions (12, 91–94). These findings are salient to healthcare professionals’ focus on social determinants of health, which can shape perinatal mental health outcomes among Hispanic and Latina women. Factors such as limited access to healthcare, economic insecurity, language barriers, immigration-related stress, and systemic racism all contribute to increased vulnerability and may influence the utilization of these interventions (24, 26). Addressing these upstream inequities alongside individual-level interventions is essential for improving maternal mental health outcomes in a sustained and equitable manner. Mental health and healthcare practitioners can capitalize on the opportunity to explore these interventions in future endeavors, such as how these non-pharmacological or self-management interventions can be sustained long-term with the hopes of ameliorating perinatal mental health. This is especially significant given the prevalence and scope of depression, anxiety, and/or stress symptoms associated with PMADs and the limited exploration of these interventions within the past twenty-five years.

## Conclusion

Our scoping review aimed to identify and describe non-pharmacological or self-management interventions associated with preventing or reducing symptoms such as depression, anxiety, and/or stress associated with PMADs in Hispanic and Latina women. We identified four interventions using principles of CBT, CBSM, mindfulness, and relaxation that have been investigated in studies using samples of predominantly Hispanic and Latina women. Future studies are warranted to further explore the effectiveness of these interventions among Hispanic and Latina perinatal women.
