# Supplementary material for: Scoping review of non-pharmacological or self-management interventions for perinatal mood and anxiety disorders tested with Hispanic and Latina women
Source: Front Psychiatry. 2025 Dec 5;16:1644798. doi: 10.3389/fpsyt.2025.1644798 (PMC12715605; doi:10.3389/fpsyt.2025.1644798)
Supplement: Supplementary file 1 [file Supplementaryfile1.docx]

**Table S1.** Search Strategy

**Concepts Searched with Corresponding Lines:**

- PMADs/Depression/Anxiety: Lines 1-13
- Hispanic/Latina Mothers: Lines 14-23
- Mind-Body Therapies/Self-Management/Exercise (Non-Pharmacological Interventions): Lines 25-31

| 1 | (PMAD or PMADS or Postpartum Mood Anxiety Disorder* or PERINATAL Mood Anxiety Disorder*).ab,ti. |
| --- | --- |
| 2 | exp Stress, Psychological/ or exp Anxiety/ or Depression/ or Depression, Postpartum/ or exp Psychological Distress/ |
| 3 | exp Mood Disorders/ or exp Anxiety Disorders/ |
| 4 | (Mood or Moodiness or Moody or Affective Disorder*).ab,ti. |
| 5 | (cardiac neuroses or cardiac neurosis or cardiac phobia or effort syndrome or hyperkinetic heart syndrome or neurocirculatory asthenia or neurocirculatory asthenias).ab,ti. |
| 6 | (neuroses or neurotic disorder or neurotic disorders or psychoneuroses).ab,ti. |
| 7 | (anankastic personalities or anankastic personality or obsessive compulsive disorder or obsessive compulsive disorders or obsessive compulsive neuroses or obsessive compulsive neurosis).ab,ti. |
| 8 | (panic attack or panic attacks or panic disorder or panic disorders).ab,ti. |
| 9 | (claustrophobia or phobia or phobias or phobic disorder or phobic disorders or phobic neuroses or scolionophobia).ab,ti. |
| 10 | (agoraphobia or crowds phobia or open spaces phobia).ab,ti. |
| 11 | (kinesiophobia or kinesophobia or kinetophobia or movement fear or movement phobia or pain related activity avoidance).ab,ti. |
| 12 | (Anxiety or Anxiet* or Anxious* or hypervigilance or Distress* or Depressive or Depression* or Stress* or angst or nervousness or post natal dysphoria or post partum dysphoria or postnatal dysphoria or postpartum dysphoria).ab,ti. |
| 13 | 1 or 2 or 3 or 4 or 5 or 6 or 7 or 8 or 9 or 10 or 11 or 12 |
| 14 | exp "Hispanic or Latino"/ |
| 15 | (hispanic* or hispanic american* or hispano* or latine* or latina* or latin or latinu* or latino* or latinx* or latin american* or Spanish speak* or mexico* or cuban* or peruvian* or dominican* or brazilian* or central american* or costa rican* or guatemalan* or honduran* or uruguayan* or Argentine* or Argentinian* or Argentinean* or panamanian* or salvadorean* or salvadoran* or salvadorian* or nicaraguan* or south america* or bolivian* or chilean* or colombian* or ecuadorian* or paraguay* or venezuelan* or puerto rican* or puerto rico* or spanish america* or boricua* or chicana* or chicano* or hispano* or hispanic american* or latino american* or spanish caribbean* or mexican american* or Mexican*).ab,ti. |
| 16 | 14 or 15 |
| 17 | Peripartum Period/ or exp Postpartum Period/ or Pregnancy/ or Parents/ or exp Mothers/ |
| 18 | (Postpartum or Post partum or Antenatal or Ante natal or Perinatal or Peri natal or Postnatal or Post natal or Puerperium or Postpartal or Post partal or Peripartum or Peri partum).ab,ti. |
| 19 | (Pregnanc* or Pregnant or Mother* or Mom or Moms or Parent*).ab,ti. |
| 20 | (Nursing adj3 Women).ab,ti. |
| 21 | (Lactating adj3 Women).ab,ti. |
| 22 | \| 17 or 18 or 19 or 20 or 21 \|  \| \| --- \| --- \| |
| 23 | 16 and 22 |
| 24 | 13 and 23 |
| 25 | exp Self-Control/ or exp Self Care/ or exp Self-Management/ |
| 26 | (Self Control or Self Regulation or Emotional Control or emotion self regulation or emotional regulation or self care or self management).ab,ti. |
| 27 | Mind-Body Therapies/ or Aromatherapy/ or Biofeedback, Psychology/ or Neurofeedback/ or Breathing Exercises/ or Qigong/ or Meditation/ or Relaxation Therapy/ or Tai Ji/ or Yoga/ or exp Exercise/ or exp Mindfulness/ |
| 28 | (Exercis* or Stretching or Walk* or Physical Activit* or Physical Conditioning or Training or Running or Jogging or Swimming or Stair Climbing or Physical Exertion).ab,ti. |
| 29 | (aroma therapies or aroma therapy or AROMATHERAP* or mind body medicine or mind body therapies or mind body therapy or mind body or bio feedback* or biofeedback or biofeedbacks or myofeedback or myofeedbacks or psychophysiologic feedback* or myo feedback* or psycho physiologic feedback* or breathing exercises or RESPIRATORY muscle training or BREATHING EXERCISE or ch'i kung or qi gong or meditation* or meditative or relaxation ecotherapies or ecotherapy or nature therapies or nature therapy or chi, tai or t'ai chi or tai chi or tai chi chuan or tai ji or tai ji quan or yoga or self compassion or Self Forgiveness or mindfulness).ab,ti. |
| 30 | ("Mamá, te entiendo" or "Stress Management and Relaxation Training for Moms" or "Centering Pregnancy with Mindfulness prenatal program" or "Mothers and Babies Course").ab,ti. |
| 31 | 25 or 26 or 27 or 28 or 29 or 30 |
| 32 | 24 and 30 |
| 33 | limit 31 to (english or spanish) |

**Table S2.** Studies of Interventions for PMADs Tested with Hispanic and Latina Women

| **Author(s)/Year of publication** | **Study Design/ Purpose** | **Country of Origin** | **Intervention** | **Intervention Group** | **Control Group** | **Cultural Adaptation** | | **Population and Follow-Up** | **Ethnicity/ Demographics** | **Outcome Code and (Measures)** |
| --- | --- | --- | --- | --- | --- | --- | --- | --- | --- | --- |
| Barrera, Wickam, & Munoz, 2015 (57) | RCT  Reduction of depressive symptoms | 23 countries worldwide | CBT | MBC, Internet based, Spanish adaptation of MBC, 8 lessons in sequential order, unlimited visits. Self-help approach (no human assistance) (*n*=57) | Single login to access an electronic version of PPD informational brochure (*n*=54) | + | | Pregnant women (mean=19.9weeks, SD=10.36), who were interested in using the Internet site, absence of MDEs, and who provided sufficient postpartum depression assessment data; follow-up through 6 months postpartum | Chile 18.9%, Spain 16.2%, Argentina 11.7%, Mexico 10.8%, Colombia 6.3%, U.S. 5.4%,  Peru 4.5%,  UK 4.5%,  others <5%  56% Married  70.6% employed full or part-time  >86% some college or degree, 13.4% with 12 yrs of education or less | B (EPDS, MDE Screener) |
| Franco, Olhaberry, Kelders, Muzard, & Cuijpers, 2024 (56) | RCT (mixed methods)    Reduction of depression symptoms | Chile | CBT | Mamá, te entiendo web app 8-week guided, individualized feedback from a clinical psychologist (*n*=33) | Usual care ("waitlist") uses of standard mental health services (*n*=32) | | ^ | Postpartum women (between 1 and 7 months) with minor or major perinatal depression; follow-up at 8- and 12-weeks post-baseline | Chilean (93.8%)  Average age 32.5 years (SD=3.8)    81.5% university education    81.5% employed | B (PHQ-9, EPDS) |
| Le, Perry, & Stuart, 2011 (47) | RCT    Prevention of depression symptoms | Wash., DC | CBT | MBC, 8 weekly 2-hour psychoeducational group sessions teach mood regulation skills; three booster sessions (6 weeks, 4 and 12 months postpartum) (*n*=112) | Usual care received information on local social services upon request (*n*=105) | | + | Healthy, low-risk pregnant women </=24 weeks gestation at risk of depression; follow-up period up to 12 months postpartum | Immigrants (54.4% El Salvador, 11.1% Honduras, 10.1% Guatemala, and 15.7% Mexico) 8.7% U.S.  90% annual household income < $30,000 | B (CES-D, BDI-II, Mood Screener) |
| Le, Perry, Genovez, & Cardeli, 2013 (48) | Qualitative (secondary data analysis from Le, Perry, & Stuart 2011)    Explore findings from the RCT parent study | Wash., DC | CBT | N/A | N/A | | ^, + | Approximately 18 months postpartum; MBC completers (at least 4 of 8 classes) (*n*=16), MBC non-completers (less than 4 classes) (*n*=8), and usual care group (*n*=15) | Average age 27.82 years (SD=4.88)    < nine years of education (M=8.52, SD=4.07)    61.5% married/partner | B (depression and MDEs) |
| Le, Perry, Grest, Genovez, Lieberman, Ortiz-Hernandez, & Serafini, 2021 (49) | Non-randomized, uncontrolled trial (mixed methods)    Prevention of depression symptoms | Wash., DC | CBT | Phase I: MBC, 6 weekly classes (*n*=86)    Phase II: Randomly selected subsample from Phase I (Qualitative interviews to understand the mechanisms and impact of participants' experiences with the intervention and study) (*n*=26) | None | | ^, + | Pregnant or postpartum women (up to 1 year) at high risk for depression; considered participants if completed three interviews at baseline (pre-intervention); follow-up 6 weeks after baseline (post-intervention), and 3 months after the 2nd interview (*n*=86) | 76.7% immigrants from Central America  Average age 27.7 years (SD=5.5)    Average 9.5 years of education (SD=4.0)    72.1% married/partner    Monthly income between $802 and $992    Average years of education 9 to 9.8 | B (PDSS-SF) |
| Muñoz, Le, Ippen, Diaz, Urizar, Soto, Mendelson, Delucchi, & Lieberman, 2007 (50) | RCT    Prevention of MDEs | California | CBT | MBC, 12 weekly small group prenatal program classes led by a facilitator supervised by a licensed clinical psychologist; 4 booster sessions at 1, 3, 6, and 12 months postpartum (*n*=21) | Usual care during the 12 weeks received routine medical care and received locally available social services information upon request (*n*=20) | | ^, + | Healthy, low-risk pregnant women between 12- and 32-weeks’ gestation at high risk of MDEs; follow-up 12 weeks after baseline assessment and at 1, 3, 6, and 12 months postpartum | 70% Latina women from Mexico/Central America;    Average age 25 years (SD=4.4)    100% < 12 years of education (M=10, SD=2.9)    76% married/partner    73% unemployed    87% annual household income < $30,000 | C (CES-D, EPDS), A (Mood Screener) |
| Urizar & Muñoz, 2011 (53) | RCT    Prevention of perceived stress and depression | California | CBT | MBC, 12 weekly small group prenatal program classes led by a facilitator supervised by a licensed clinical psychologist; 4 booster sessions at 1, 3, 6, and 12 months postpartum (*n*=24) | A usual care group (*n*=33) (high risk of prenatal depression); Low-risk comparison group (*n*=29); both groups- routine medical care and received local social services information upon request or if developed clinical depression | | ^ | Healthy, low-risk pregnant women between 6- and 28-weeks’ gestation at high risk of depression; follow-up at 6 and 18 months postpartum | 80% Latina from Mexico/Central America  Age range 18 to 35 years (average 25 years)    Majority not complete high school    77% married/partner    87% annual household income < $30,000 | C (CES-D), (use perceived stress visual analog scale one-item at 18 months postpartum); PANAS |
| Ponting, Chavia, Schetter, & Urizar, 2022 (54) | RCT    Reduction of anxiety symptoms | California | CBSM | SMART Moms, 8 weekly 2-hour group sessions led by a facilitator with coping and relaxation skills (*n*=55) | Active control received weekly prenatal health information via mail (*n*=45) | | ^ | Healthy, low-risk less than 17 weeks gestation pregnant women with subclinical anxiety (*n*=100); follow-up 8 weeks after baseline, at 30 to 32 weeks gestation, and 3 months postpartum | 74% Latina; 18% Black;  58.2% CBSM and 55.6% control individuals not born in the U.S.; 87.7% Mexico  Average age 26.5 years (SD=.9)    75.2% annual household income < $20,000  70.3% unemployed  47.3% CBSM and 51.1% control married/partner    62.4% high school or less | C (PrAS)- 30-32 weeks’ gestation, (subscale of the STPI-SF)-30-32 weeks gestation and 3 months postpartum |
| Urizar, Caliboso, Gearhart, Yim, & Schetter, 2019 (51) | RCT    Process evaluation of CBSM program: association between implementation fidelity and outcomes (Urizar, Yim, et al., 2019) | California | CBSM | SMART Moms, 8 weekly prenatal classes teaching coping and relaxation skills for stressors and daily challenges experienced during pregnancy and motherhood (*n*=55) | Active control in an 8-week program received printed educational materials (*n*=45) | | ^ | Healthy, low-risk less than 17 weeks gestation experiencing low or high anxiety; follow-up during 2nd and 3rd trimesters and 3 months postpartum | CBSM group statistics available:  71% Latina:  57% born outside the U.S., 70% in Mexico; 14.5% African American, 5.5% Asian American, 7.3% Non-Hispanic White, 3.6% mixed ethnicity  Average age 26.3 years (SD=6.4)    47.3% married    72.7% unemployed    67.3% high school or less    76% annual household income < $20,000 | A (perceived stress, 5-point Likert evaluation, PANAS-SF) |
| Urizar, Yim, Rodriquez, & Schetter, 2019 (52) | RCT    Reduction of perceived stress | California | CBSM | SMART Moms, 8 weekly prenatal classes teaching coping and relaxation skills for stressors and daily challenges experienced during pregnancy and motherhood (*n*=55) | Active control in an 8-week program received printed educational materials (*n*=45) | | ^ | Healthy, low-risk less than 17 weeks gestation low-income pregnant women experiencing low or high anxiety; follow-up during 2nd and 3rd trimesters and 3 months postpartum | 71% Latina:  57% were born outside the U.S., 70% from Mexico; 29% non-Latina (18% African American)  Average 27 years of age (SD=6.26    51% single    70% unemployed    76% annual household income < $20,000    71% high school education or less | A (PSS-14) |
| Duncan, Zhang, Santana, Cook, Castro-Smyth, Hutchison, Huynh, Mallareddy, Jurkiewicz, & Bardacke, 2023 (46) | Non-RCT    Reduction of depression, stress, and anxiety symptoms | California | MBI | Centering Pregnancy with Mindfulness prenatal program 9 weekly 3-hour sessions (mindfulness meditation 30 minutes/day, 6 days/week for 9 weeks) (*n*=25) | Active control Centering Pregnancy prenatal program with 10 2-hour sessions (*n*=24) | | ^, + | Healthy, low-risk less than 3rd trimester; follow-up during 3rd trimester and 6 to 20 weeks post final centering session | 65.3% Latina/Latin American/Hispanic; 65% born outside the U.S.  Average age 26 years    73.4% completed high school or less    61.2% annual household income < $30,000    4% LGBTQ+, 90% Black, Indigenous, and People of Color (65% Latina/e/x), 10% White | A (STAI), (EPDS); B (PSS-10, CES-D) |
| Primo & Amorim, 2008 (55) | Non-RCT    Prevention of anxiety symptoms | Brazil | R | Non-manualized relaxation techniques (quiet setting, mental device, passive attitude, comfortable position) twice/day 2 consecutive days (*n*=30) | Usual care (no details provided) (*n*=30) | | ^ | Healthy inpatient postpartum women (48 to 72 hours of delivery) with term deliveries; follow-up 1 week postpartum | Age range 19 to 25 years    Majority did not complete high school    73.3% control group and 66.7% intervention group married/partner | A (STAI) |

*Notes*:

BDI-II = Beck Depression Inventory- Second Edition; CBSM = cognitive behavioral stress management; CBT = cognitive behavioral therapy; CES-D = Center for Epidemiologic Studies Depression Scale; EPDS = Edinburgh Postnatal Depression Scale; EPDS-US = Edinburgh Postnatal Depression Scale; MBC = Mothers and Babies Course; MBI = mindfulness based intervention; MDEs = major depressive episodes; MINI = Mini International Neuropsychiatric Interview; PANAS = Positive and Negative Affect Score; PAS = Prenatal Anxiety Scale; PDSS-SF = Postpartum Depression Screening Scale- Short Form; PHQ-9 = Patient Health Questionnaire-9; PPD = postpartum depression; PrAS = pregnancy-related anxiety symptoms; PSS-10 = Perceived Stress Scale-10; PSS-14 = Perceived Stress Scale-14; R = relaxation; RCT = randomized controlled trial; SMART Moms = Stress Management and Relaxation Training for Moms; STAI = State-Trait Anxiety Inventory; STPI-SF = State-Trait Personality Index-Short Form; U.S. = United States; Wash., DC = Washington, DC

*Cultural Adaptations*:

^ Spanish translation available

+ Culturally and linguistically tailored intervention (cultural congruence)

*Outcomes Codes*:

A. Intervention effective (reduction of symptoms or major depressive episodes) in the intervention group

B. Intervention equally as effective (reduction of symptoms or major depressive episodes) as in the control group

C. Intervention not effective (no reduction of symptoms or major depressive episodes)

**Table S3.** Summary of Non-pharmacological or Self-management Interventions in Hispanic and Latina Women

| Cognitive Behavioral Therapy  (*n*=7 studies; *n*=645 total participants) | Cognitive Behavioral Stress Management  (*n*=3 studies; *n*=300 total participants) | Mindfulness  (*n*=1 study; *n*=49) | Relaxation  (*n*=1 study; *n*=60) |
| --- | --- | --- | --- |
| - May prevent or reduce:   - Depression symptoms   - Major depressive episodes - May not prevent:   - Stress symptoms - Interventions:   - MBC (6 studies reviewed [47, 48, 49, 50, 53, 57])   - Mamá, te entiendo (1 study reviewed [56]) | - May reduce:   - Stress symptoms - May not reduce:   - Anxiety symptoms - Intervention:   - SMART Moms (3 studies reviewed [51, 52, 54]) | - May reduce:   - Depression symptoms   - Anxiety symptoms - May not reduce:   - Stress symptoms - Intervention:   - MBCP (1 study reviewed [46]) | - May prevent:   - Anxiety symptoms - Intervention:   - Non-manualized relaxation (1 study reviewed [55]) |
